# Anticipated deaths with physician care (*mitori*) at home in one town in Japan: A preliminary report

**DOI:** 10.1002/jgf2.648

**Published:** 2023-10-03

**Authors:** Nobuyuki Kajiwara, Shinya Suezaki, Megumi Okamoto, Yasuo Kuwahara, Masanori Okui, Akiyoshi Nishimura, Hirono Takabayashi

**Affiliations:** ^1^ Toyono Town National Health Insurance Clinic Toyono Japan; ^2^ Department of Nephrology Ikeda City Hospital Ikeda Japan; ^3^ Fast Doctor Co., Ltd. Minato Japan; ^4^ Hoken‐ka, Hokenfukushi‐bu, Toyono Town Toyono Japan; ^5^ Toyono‐gun Kankyoshisetsukumiai Toyono Japan; ^6^ Okui Clinic Nose Japan; ^7^ Department of Forensic Medicine, Institute of Health Biosciences Tokushima University Graduate School Tokushima Japan; ^8^ Ikeda Public Health Center Ikeda Japan

**Keywords:** anticipated death (*mitori* [看取り]), death at home, home medical care, Japanese death certificate system (*shiboukohyou* [死亡小票]), unnatural death [異状死]

## Abstract

**Background:**

There are few reports on the numbers of anticipated deaths (*mitori* [看取り]) at home in Japan.

**Method:**

We used the Japanese death certificate system (*shiboukohyou* [死亡小票]) for Toyono town citizens who died between 2020 and 2022 and judged a death to constitute *mitori* when the certificate was not written by a doctor referred from the police.

**Results:**

Among 756 deaths, 109 (14.4%) were *mitori* at home. Deaths at home were 144 and *mitori* at home accounted for 75.7%.

**Conclusion:**

*Shiboukohyou* appear to provide numbers of *mitori* at home. Death certificate should include a space which shows *mitori* or not.

## Graphical abstract

We used the Japanese death certificate system (*shiboukohyou* [死亡小票]) for Toyono town citizens. Among 756 deaths, 109 (14.4%) were anticipated deaths (*mitori* [看取り]) at home. Deaths at home were 144 and *mitori* at home accounted for 75.7%. Death certificate should include a space which shows *mitori* or not.

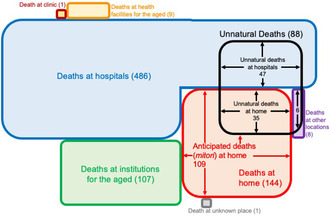

## 1 BACKGROUND

In Japan, the total number of deaths and deaths occurring at home in each municipality are publicly announced. However, deaths at home including not only individuals who were expected to die at home with physician care (end‐of‐life care at home, or *mitori* [看取り in Japanese] at home) but also those who die unexpectedly (sometimes alone) owing to sudden illnesses, asphyxia, suicides, and other causes (referred to as an unnatural death [異状死 in Japanese]).^1^ Consequently, obtaining accurate numbers of cases of *mitori* at home is difficult in Japan. Prior studies about end‐of‐life care at home in Japan focused deaths at home.^1^, ^2^ However, including unnatural deaths at home in deaths at home is methodological issue. Surveys in Japan show that many people prefer to die at home.^3^ Despite this, many people die in hospitals. Toyono town has an altitude of 150–600 m above sea level and a population of 18,451 as of February 1, 2023.^4^ There is no hospital. The percentage of residents over the age of 65 is 48.4%, which is substantially higher than the Japanese average of 29.1%.^5^ There is a need to ascertain the extent of *mitori* at home for evaluating the end‐of‐life care in this town. The aims of this study were to inform medical care planning in Toyono town and estimate the number of cases of *mitori* at home among the reported deaths at home in other areas. To achieve this, we decided to use closed data from the Japanese death certificate system (*shiboukohyou*, [死亡小票 in Japanese]).

## 2 METHODS

We utilized secondary data from the *shiboukohyou* which includes death certificates [死亡診断書 in Japanese] and autopsy certificates (death certificates by postmortem diagnosis) [死体検案書 in Japanese] of Toyono town residents who died between January 1, 2020 and December 31, 2022. These data were provided by the Ministry of Health, Labour and Welfare, and with their permission, we accessed the data at March 2023. The analyzed items were date of death, date of birth, gender, place of death, cause of death, duration of cause of death, and the name and address of the doctor who wrote the death or autopsy certificate.

We determined a death to constitute an unnatural death when its certificate was written by a doctor referred by the police or affiliated with the forensic medicine department at a medical college. We considered all deaths, except for unnatural deaths, as cases of *mitori*. We then verified the cause of death and the duration of the cause of death to evaluate the accuracy of this classification.

## 3 RESULTS

**FIGURE 1:**
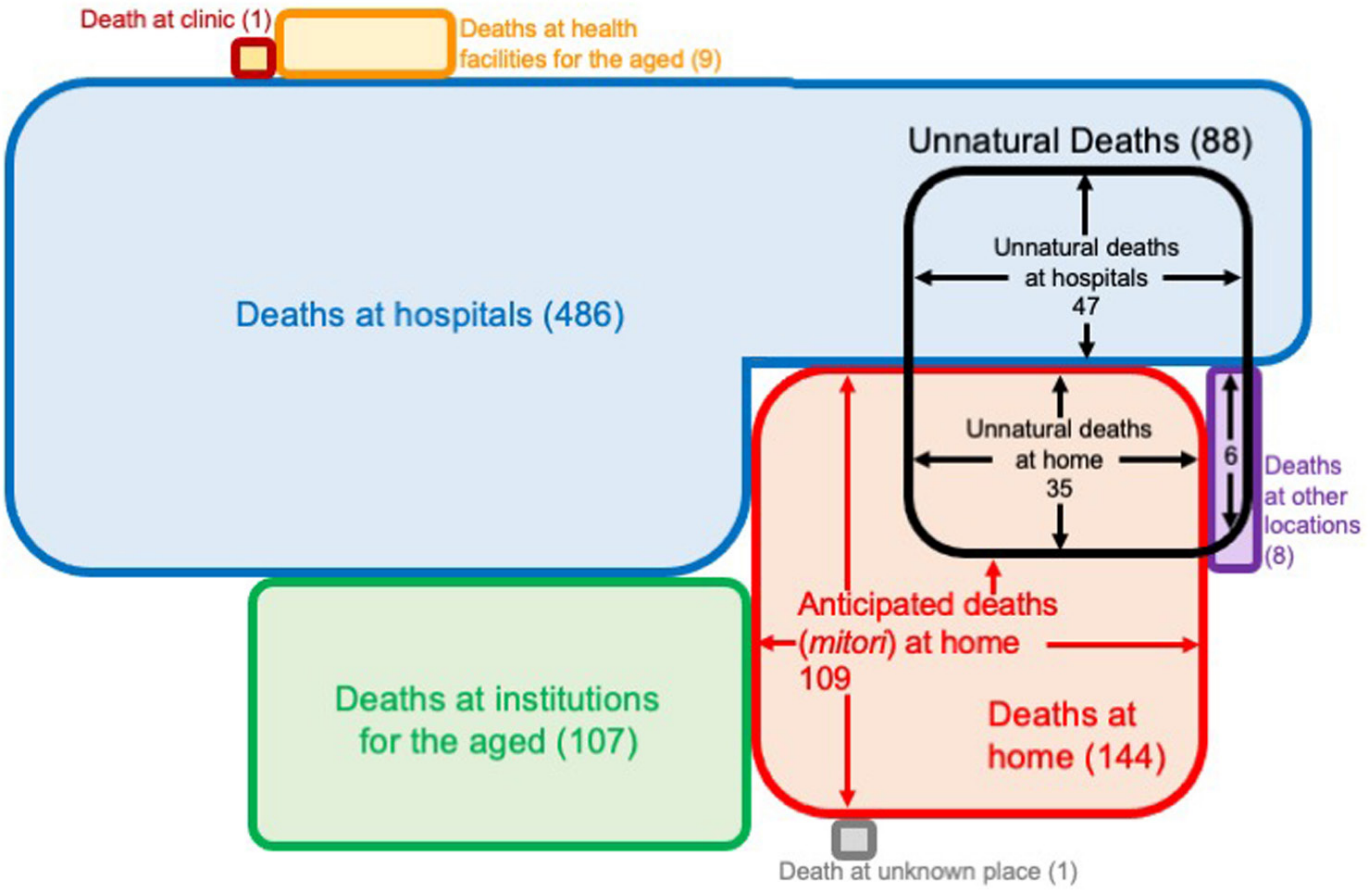
Place of death and unnatural death. Areas of quadrilaterals indicate approximate numbers of deaths. Unnatural deaths occurred at hospitals, homes, and the other places.

There were 756 recorded deaths among Toyono town residents between 2020 and 2022. The places of death were hospitals [病院 in Japanese] (486; 64.3%), clinic [診療所 in Japanese] (1; 0.1%), health facilities for the aged [介護老人保健施設 in Japanese] (9; 1.2%), maternity hospital [助産院 in Japanese] (0; 0%), institutions for the aged [老人ホーム in Japanese] (107; 14.2%), homes [自宅 in Japanese] (144; 19.0%), other locations [その他 in Japanese] (8; 1.1%), and unknown place [不明 in Japanese] (1; 0.1%) (Figure 1). Unnatural deaths were 88. Places of death of them were 47 at hospitals, 35 at homes, and 6 at other locations (Figure 1). There were 109 cases of *mitori* at home, accounting for 75.7% of deaths at home. When adding the number of unnatural deaths that occurred at hospitals, which might otherwise have occurred as deaths at home, to the number of deaths at home as a denominator, the rate of cases of *mitori* at home among deaths at home fell to 57.1% (Table 1). We examined the cause of death and the duration of the cause of death for each subject and confirmed the appropriateness of their classification as cases of *mitori* or unnatural deaths.

**TABLE 1:** Rates of anticipated deaths at home with physician care (*mitori* at home).

Year	2020	2021	2022	Total or average
*Mitori* at home	33	36	40	109
Deaths at home	42	48	54	144
(*Mitori* at home/deaths at home) * 100 [%]	78.6	75.0	74.1	75.7
Deaths at home + unnatural deaths at hospitals	54	66	71	191
{*Mitori* at home/(deaths at home + unnatural deaths at hospitals)} * 100 [%]	61.1	54.5	56.3	57.1
Total deaths	227	261	268	756
(*Mitori* at home/total deaths) * 100 [%]	14.5	13.8	14.9	14.4

*Note*: “*Mitori* at home” means anticipated deaths at home wherein physicians had provided care during home visits. “Unnatural deaths” means unexpected deaths because of sudden illnesses, traffic accidents, suicides, and other causes.

## 4 DISCUSSION

In the present study, we ascertained the number of cases of *mitori* at home in Toyono town, which was previously unknown. There are only two Japanese reports using data from *shiboukohyou* with regard to numbers of cases of *mitori* at home, with one examining data from Nerima city in the Greater Tokyo Area, and the other examining data from Kashiwa city in Chiba Prefecture.^6^, ^7^ Cases of *mitori* at home accounted for 6.5%–16.2% of total deaths in Nerima city between 2011 and 2021, and 4.5%–8.0% in Kashiwa city between 2011 and 2018. One factor influencing these differences may be the lower accessibility to hospitals in Toyono town. The shortage of hospital beds owing to the surge of patients with COVID‐19 is another possible explanatory factor.

The proportion of cases of *mitori* at home among deaths at home was 75.7% in Toyono town, while that for Nerima city was reportedly 42.2%–65.1% between 2011 and 2021, and that for Kashiwa city was 32.1%–53.2% between 2011 and 2018. However, when we added the number of unnatural deaths at hospitals to the number of deaths at home as a denominator (assuming many such deaths would have otherwise occurred at home), the proportion decreased to 57.1%. When a patient dies at home or is transported by ambulance to a hospital while close to death, the classification of the death as a death at home or one at a hospital is left up to the discretion of the doctor referred by the police. Given the above, the proportion of cases of *mitori* at home among deaths at home should be estimated with a wide margin of 30%–80%. Because the proportions vary in Nerima city, Kashiwa city, and Toyono town, it is difficult to estimate the number of *mitori* at home based on the number of deaths at home.

If the same doctor provides both home care medicine for *mitori* at home and conducts autopsies referred by the police, then it is necessary to review each individual case and judge whether it is *mitori* or an unnatural death. This is a limitation of the present method. Judging that a death is *mitori* or an unnatural death by a doctor who write a death or autopsy certificate is accurate. Adding a space clearly indicating classification as *mitori* or an unnatural death in death and autopsy certificates and making public those data would be useful for informing measures for end‐of‐life care.

In the future, we hope to examine the differences in the rates of *mitori* at home by region or by the cause of death. In addition, we hope to examine the rate of *mitori* at home in more broad areas.

## 5 CONCLUSION

The number of cases of *mitori* at home in Toyono town was apparent upon analysis of *shiboukohyou* data. It accounted for 14.4% of all deaths in the town between 2020 and 2022. It is difficult to estimate the number of *mitori* at home from the number of deaths at home. Death certificate should include a space which shows *mitori* or not.

## AUTHOR CONTRIBUTIONS

Nobuyuki Kajiwara drafted the manuscript. Nobuyuki Kajiwara and Shinya Suezaki analyzed the data. All authors contributed to the manuscript preparation and agree to be held accountable for the present report.

## FUNDING INFORMATION

This research did not receive any specific grant from funding agencies in the public, commercial, or not‐for‐profit sectors.

## CONFLICT OF INTEREST STATEMENT

The authors declare that they have no conflict of interest.

## ETHICS STATEMENT

This study received approval for the secondary utilization of inquiry reports in accordance with the Statistics Act of Japan. We received permission from the Ministry of Health, Labour and Welfare under the 33rd article of the Statistics Act and related laws (Kouseiroudousyouhatsuseitou 0222 dai1gou [厚生労働省発政統 0222 第1号 in Japanese]). This study is an observational study not involving any intervention and was approved by the ethics committee of Ikeda City Hospital (reference number: 3480).

## SUPPLEMENTARY NOTE

We made an independent investigation from prompt data. Final data in a census of the population are made public in the website of the Ministry of Health, Labour and Welfare (https://www.mhlw.go.jp/toukei/list/81‐1b.html#01).
